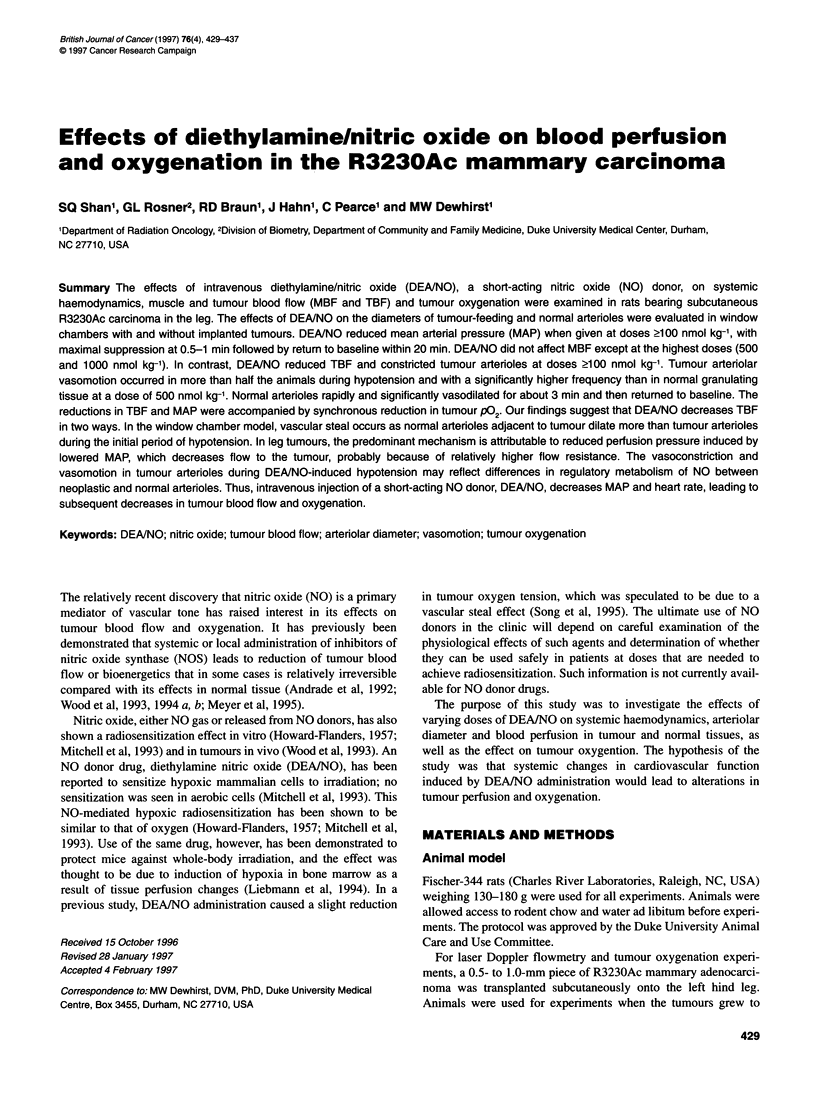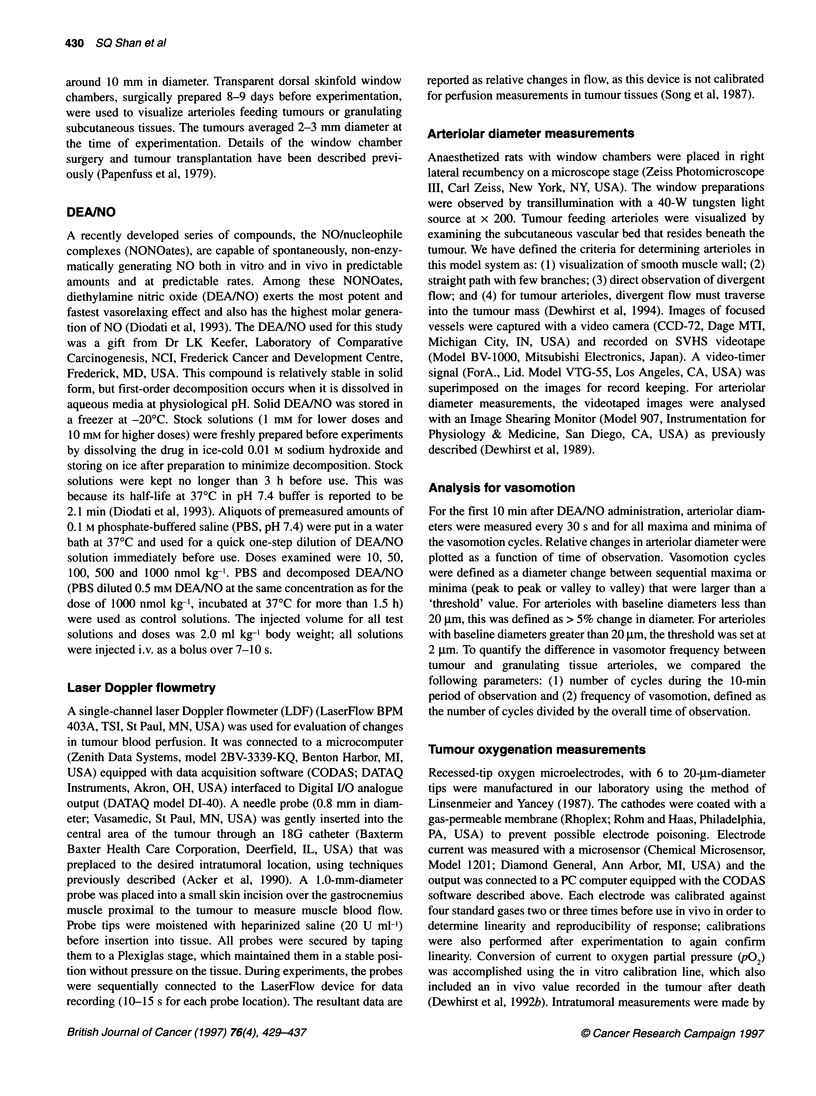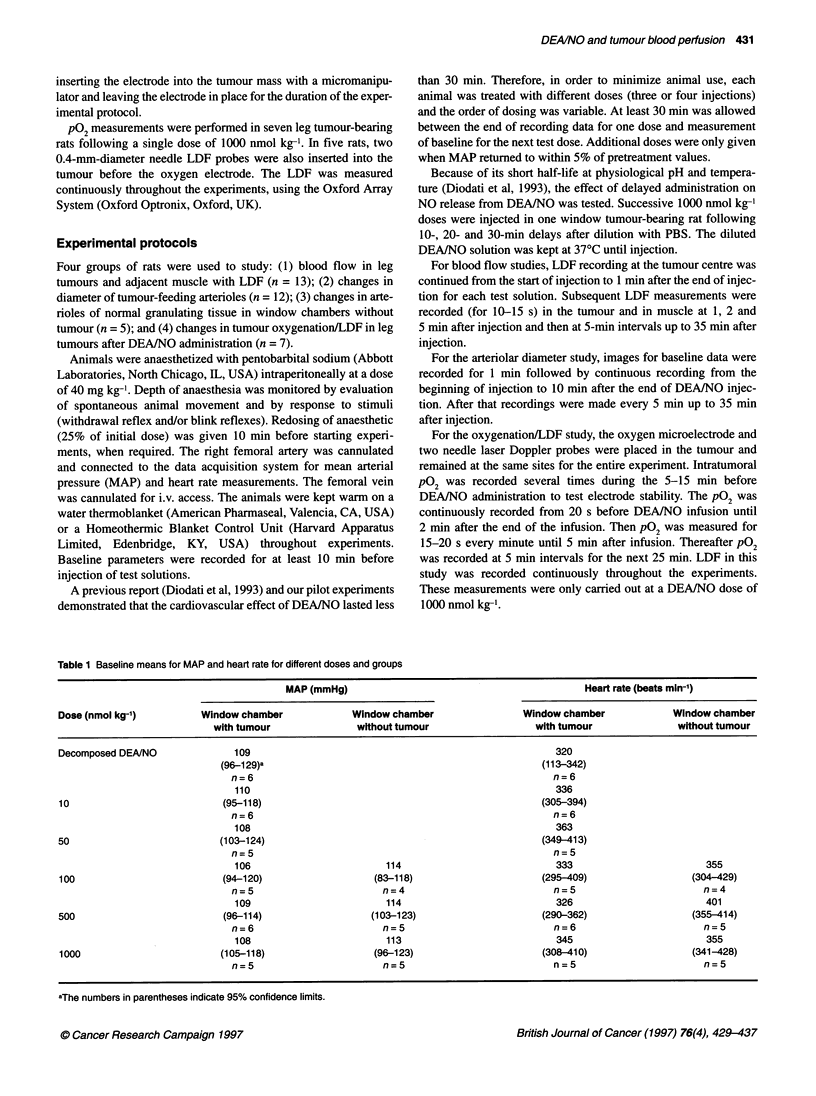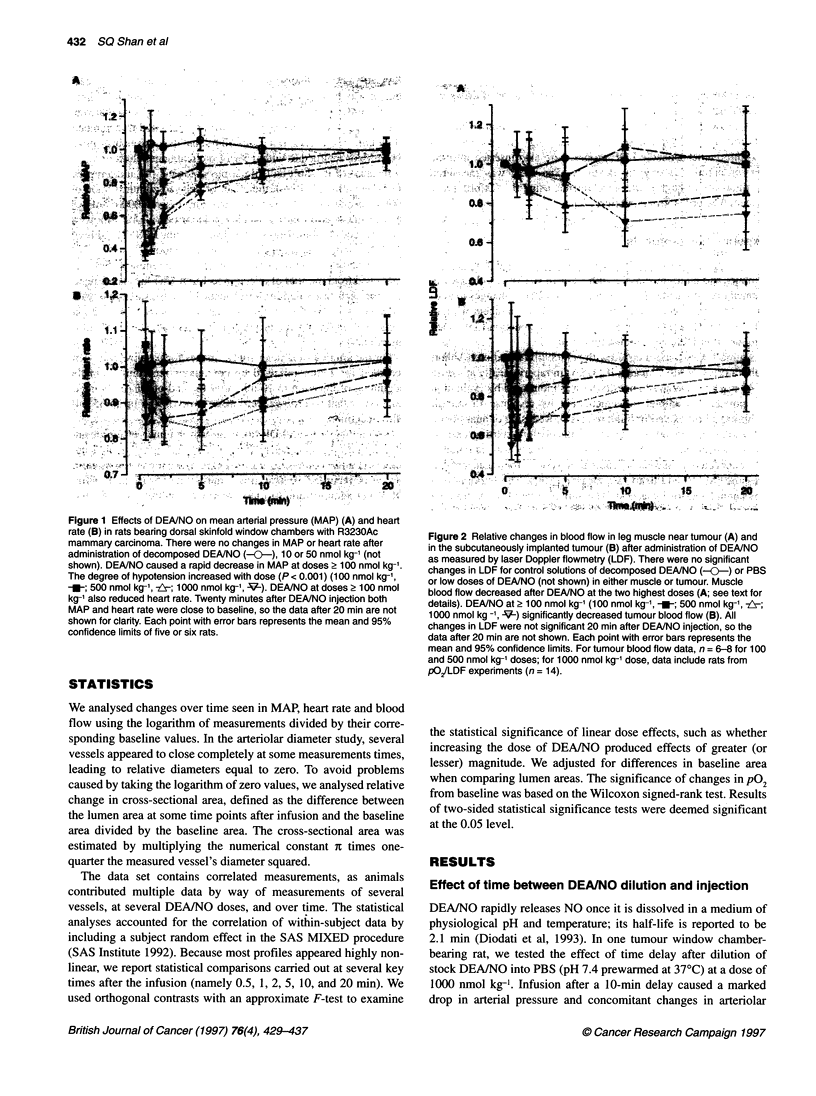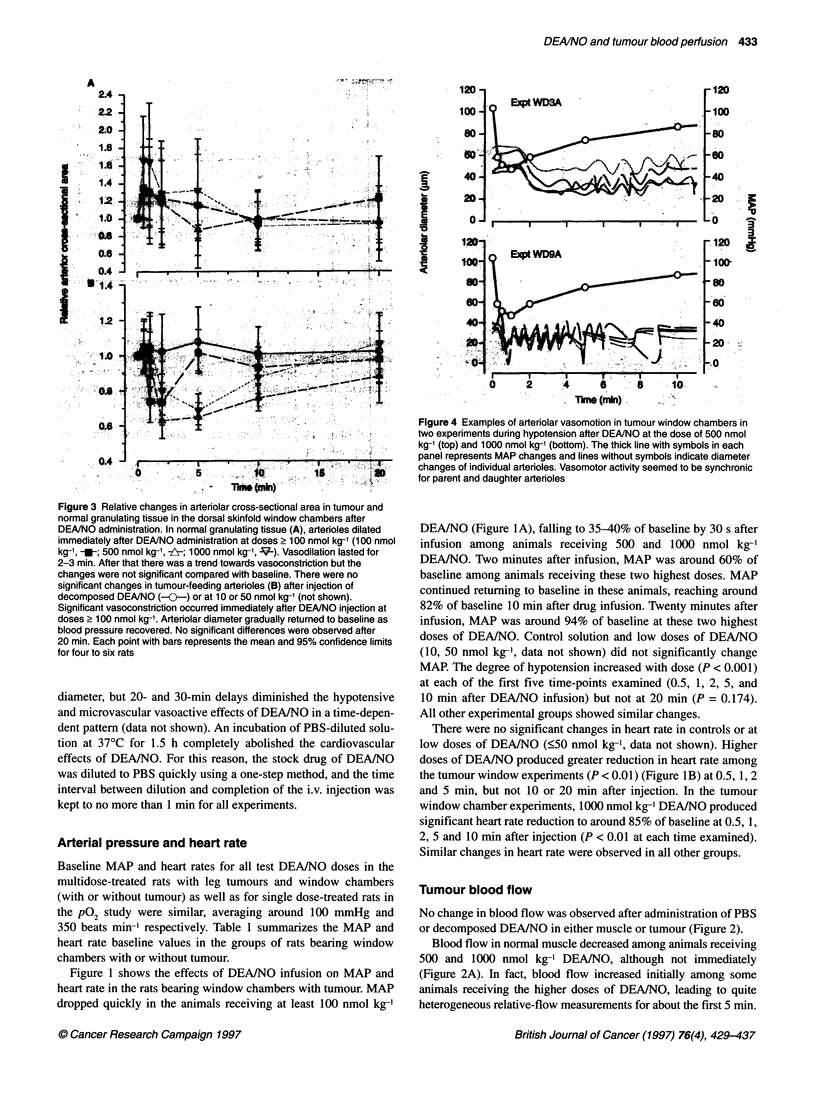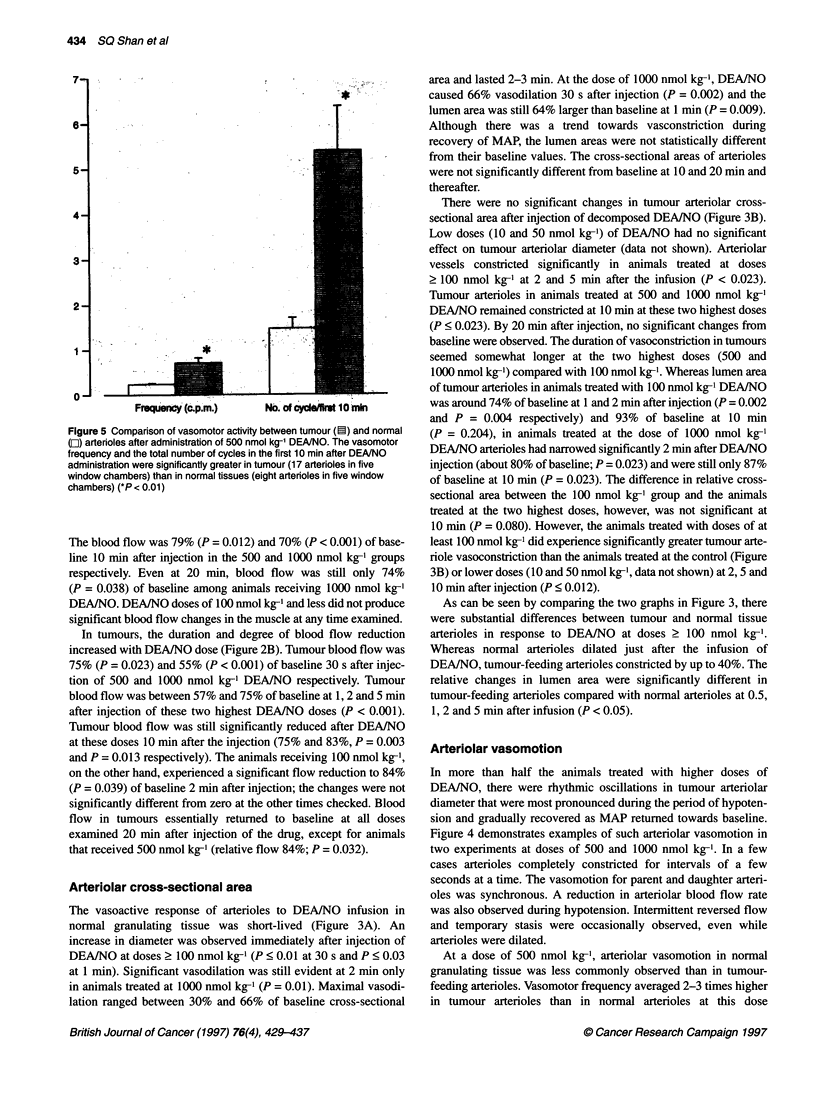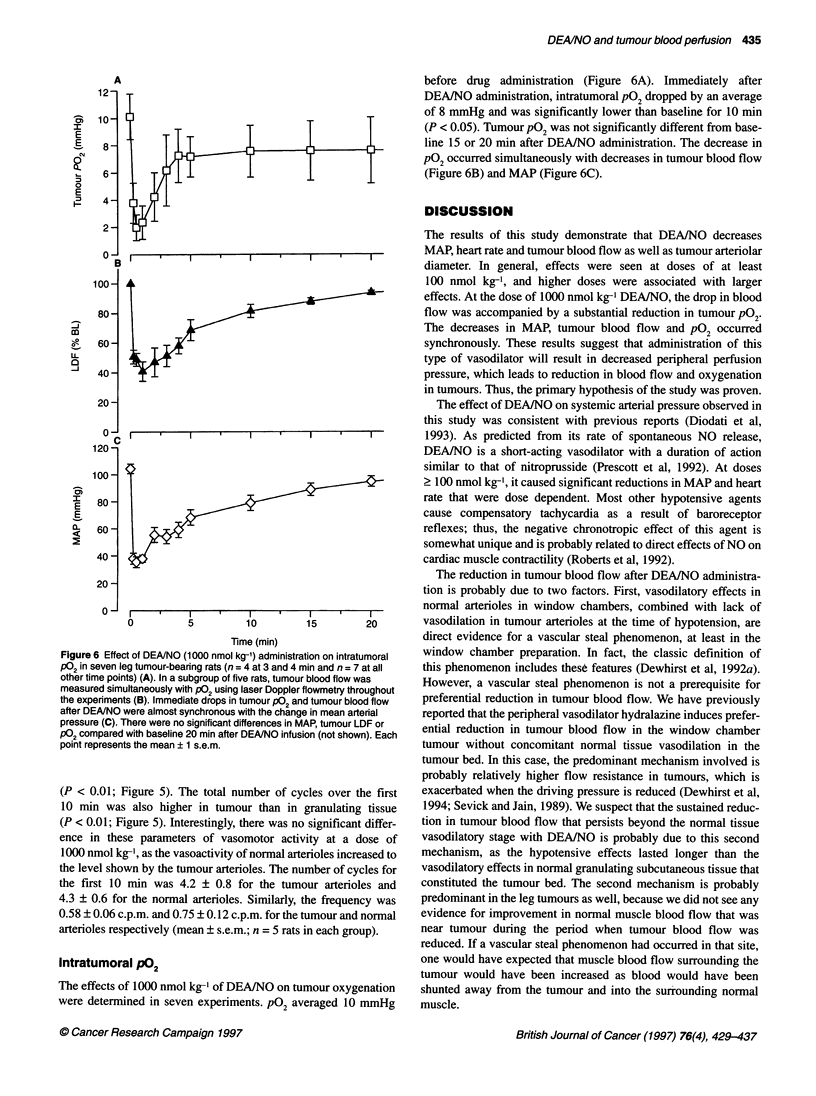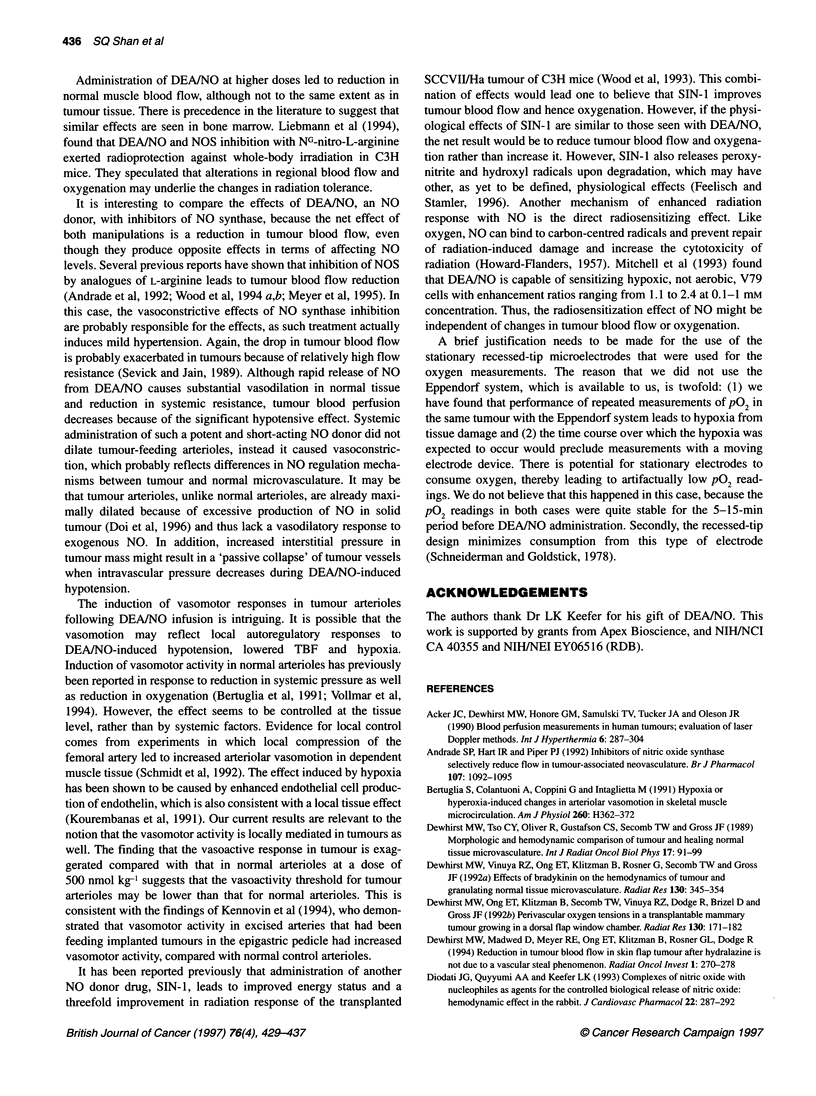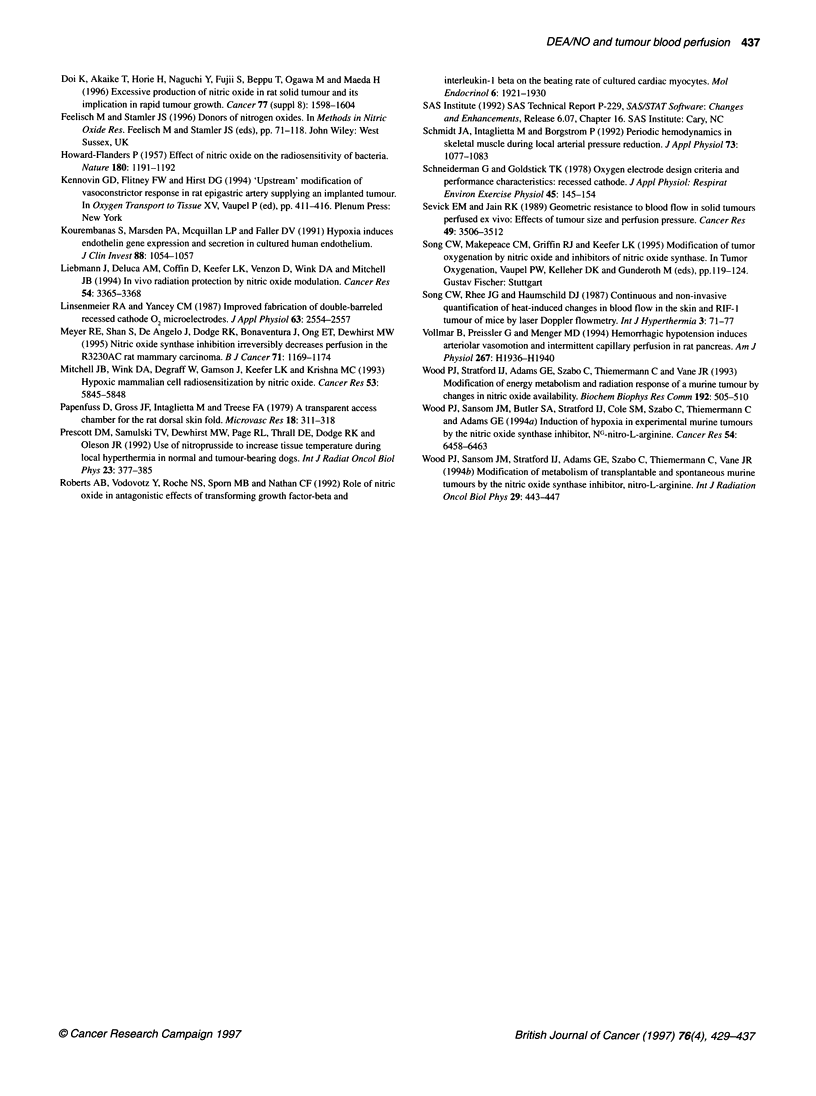# Effects of diethylamine/nitric oxide on blood perfusion and oxygenation in the R3230Ac mammary carcinoma.

**DOI:** 10.1038/bjc.1997.406

**Published:** 1997

**Authors:** S. Q. Shan, G. L. Rosner, R. D. Braun, J. Hahn, C. Pearce, M. W. Dewhirst

**Affiliations:** Department of Radiation Oncology, Duke University Medical Center, Durham, NC 27710, USA.

## Abstract

The effects of intravenous diethylamine/nitric oxide (DEA/NO), a short-acting nitric oxide (NO) donor, on systemic haemodynamics, muscle and tumour blood flow (MBF and TBF) and tumour oxygenation were examined in rats bearing subcutaneous R3230Ac carcinoma in the leg. The effects of DEA/NO on the diameters of tumour-feeding and normal arterioles were evaluated in window chambers with and without implanted tumours. DEA/NO reduced mean arterial pressure (MAP) when given at doses > or = 100 nmol kg(-1), with maximal suppression at 0.5-1 min followed by return to baseline within 20 min. DEA/NO did not affect MBF except at the highest doses (500 and 1000 nmol kg(-1)). In contrast, DEA/NO reduced TBF and constricted tumour arterioles at doses > or = 100 nmol kg(-1). Tumour arteriolar vasomotion occurred in more than half the animals during hypotension and with a significantly higher frequency than in normal granulating tissue at a dose of 500 nmol kg(-1). Normal arterioles rapidly and significantly vasodilated for about 3 min and then returned to baseline. The reductions in TBF and MAP were accompanied by synchronous reduction in tumour pO2. Our findings suggest that DEA/NO decreases TBF in two ways. In the window chamber model, vascular steal occurs as normal arterioles adjacent to tumour dilate more than tumour arterioles during the initial period of hypotension. In leg tumours, the predominant mechanism is attributable to reduced perfusion pressure induced by lowered MAP, which decreases flow to the tumour, probably because of relatively higher flow resistance. The vasoconstriction and vasomotion in tumour arterioles during DEA/NO-induced hypotension may reflect differences in regulatory metabolism of NO between neoplastic and normal arterioles. Thus, intravenous injection of a short-acting NO donor, DEA/NO, decreases MAP and heart rate, leading to subsequent decreases in tumour blood flow and oxygenation.


British Journal of Cancer (1997) 76(4), 429–437
© 1997 Cancer Research Campaign

### Effects of diethylamine/nitric oxide on blood perfusion and oxygenation in the R3230Ac mammary carcinoma

**SQ Shan[1], GL Rosner[2], RD Braun[1], J Hahn[1], C Pearce[1] and MW Dewhirst[1]**

[1]Department of Radiation Oncology, [2]Division of Biometry, Department of Community and Family Medicine, Duke University Medical Center, Durham, NC 27710, USA

**Summary** The effects of intravenous diethylamine/nitric oxide (DEA/NO), a short-acting nitric oxide (NO) donor, on systemic haemodynamics, muscle and tumour blood flow (MBF and TBF) and tumour oxygenation were examined in rats bearing subcutaneous R3230Ac carcinoma in the leg. The effects of DEA/NO on the diameters of tumour-feeding and normal arterioles were evaluated in window chambers with and without implanted tumours. DEA/NO reduced mean arterial pressure (MAP) when given at doses ≥100 nmol kg$^{-1}$, with maximal suppression at 0.5–1 min followed by return to baseline within 20 min. DEA/NO did not affect MBF except at the highest doses (500 and 1000 nmol kg$^{-1}$). In contrast, DEA/NO reduced TBF and constricted tumour arterioles at doses ≥100 nmol kg$^{-1}$. Tumour arteriolar vasomotion occurred in more than half the animals during hypotension and with a significantly higher frequency than in normal granulating tissue at a dose of 500 nmol kg$^{-1}$. Normal arterioles rapidly and significantly vasodilated for about 3 min and then returned to baseline. The reductions in TBF and MAP were accompanied by synchronous reduction in tumour $pO_2$. Our findings suggest that DEA/NO decreases TBF in two ways. In the window chamber model, vascular steal occurs as normal arterioles adjacent to tumour dilate more than tumour arterioles during the initial period of hypotension. In leg tumours, the predominant mechanism is attributable to reduced perfusion pressure induced by lowered MAP, which decreases flow to the tumour, probably because of relatively higher flow resistance. The vasoconstriction and vasomotion in tumour arterioles during DEA/NO-induced hypotension may reflect differences in regulatory metabolism of NO between neoplastic and normal arterioles. Thus, intravenous injection of a short-acting NO donor, DEA/NO, decreases MAP and heart rate, leading to subsequent decreases in tumour blood flow and oxygenation.

**Keywords:** DEA/NO; nitric oxide; tumour blood flow; arteriolar diameter; vasomotion; tumour oxygenation

The relatively recent discovery that nitric oxide (NO) is a primary mediator of vascular tone has raised interest in its effects on tumour blood flow and oxygenation. It has previously been demonstrated that systemic or local administration of inhibitors of nitric oxide synthase (NOS) leads to reduction of tumour blood flow or bioenergetics that in some cases is relatively irreversible compared with its effects in normal tissue (Andrade et al, 1992; Wood et al, 1993, 1994 *a, b*; Meyer et al, 1995).

Nitric oxide, either NO gas or released from NO donors, has also shown a radiosensitization effect in vitro (Howard-Flanders, 1957; Mitchell et al, 1993) and in tumours in vivo (Wood et al, 1993). An NO donor drug, diethylamine nitric oxide (DEA/NO), has been reported to sensitize hypoxic mammalian cells to irradiation; no sensitization was seen in aerobic cells (Mitchell et al, 1993). This NO-mediated hypoxic radiosensitization has been shown to be similar to that of oxygen (Howard-Flanders, 1957; Mitchell et al, 1993). Use of the same drug, however, has been demonstrated to protect mice against whole-body irradiation, and the effect was thought to be due to induction of hypoxia in bone marrow as a result of tissue perfusion changes (Liebmann et al, 1994). In a previous study, DEA/NO administration caused a slight reduction in tumour oxygen tension, which was speculated to be due to a vascular steal effect (Song et al, 1995). The ultimate use of NO donors in the clinic will depend on careful examination of the physiological effects of such agents and determination of whether they can be used safely in patients at doses that are needed to achieve radiosensitization. Such information is not currently available for NO donor drugs.

The purpose of this study was to investigate the effects of varying doses of DEA/NO on systemic haemodynamics, arteriolar diameter and blood perfusion in tumour and normal tissues, as well as the effect on tumour oxygention. The hypothesis of the study was that systemic changes in cardiovascular function induced by DEA/NO administration would lead to alterations in tumour perfusion and oxygenation.

*Received 15 October 1996*
*Revised 28 January 1997*
*Accepted 4 February 1997*

*Correspondence to:* MW Dewhirst, DVM, PhD, Duke University Medical Centre, Box 3455, Durham, NC 27710, USA

### MATERIALS AND METHODS

### Animal model

Fischer-344 rats (Charles River Laboratories, Raleigh, NC, USA) weighing 130–180 g were used for all experiments. Animals were allowed access to rodent chow and water ad libitum before experiments. The protocol was approved by the Duke University Animal Care and Use Committee.

For laser Doppler flowmetry and tumour oxygenation experiments, a 0.5- to 1.0-mm piece of R3230Ac mammary adenocarcinoma was transplanted subcutaneously onto the left hind leg. Animals were used for experiments when the tumours grew to around 10 mm in diameter. Transparent dorsal skinfold window chambers, surgically prepared 8–9 days before experimentation, were used to visualize arterioles feeding tumours or granulating subcutaneous tissues. The tumours averaged 2–3 mm diameter at the time of experimentation. Details of the window chamber surgery and tumour transplantation have been described previously (Papenfuss et al, 1979).

### DEA/NO

A recently developed series of compounds, the NO/nucleophile complexes (NONOates), are capable of spontaneously, non-enzymatically generating NO both in vitro and in vivo in predictable amounts and at predictable rates. Among these NONOates, diethylamine nitric oxide (DEA/NO) exerts the most potent and fastest vasorelaxing effect and also has the highest molar generation of NO (Diodati et al, 1993). The DEA/NO used for this study was a gift from Dr LK Keefer, Laboratory of Comparative Carcinogenesis, NCI, Frederick Cancer and Development Centre, Frederick, MD, USA. This compound is relatively stable in solid form, but first-order decomposition occurs when it is dissolved in aqueous media at physiological pH. Solid DEA/NO was stored in a freezer at –20°C. Stock solutions (1 mM for lower doses and 10 mM for higher doses) were freshly prepared before experiments by dissolving the drug in ice-cold 0.01 M sodium hydroxide and storing on ice after preparation to minimize decomposition. Stock solutions were kept no longer than 3 h before use. This was because its half-life at 37°C in pH 7.4 buffer is reported to be 2.1 min (Diodati et al, 1993). Aliquots of premeasured amounts of 0.1 M phosphate-buffered saline (PBS, pH 7.4) were put in a water bath at 37°C and used for a quick one-step dilution of DEA/NO solution immediately before use. Doses examined were 10, 50, 100, 500 and 1000 nmol $kg^{-1}$. PBS and decomposed DEA/NO (PBS diluted 0.5 mM DEA/NO at the same concentration as for the dose of 1000 nmol $kg^{-1}$, incubated at 37°C for more than 1.5 h) were used as control solutions. The injected volume for all test solutions and doses was 2.0 ml $kg^{-1}$ body weight; all solutions were injected i.v. as a bolus over 7–10 s.

### Laser Doppler flowmetry

A single-channel laser Doppler flowmeter (LDF) (LaserFlow BPM 403A, TSI, St Paul, MN, USA) was used for evaluation of changes in tumour blood perfusion. It was connected to a microcomputer (Zenith Data Systems, model 2BV-3339-KQ, Benton Harbor, MI, USA) equipped with data acquisition software (CODAS; DATAQ Instruments, Akron, OH, USA) interfaced to Digital I/O analogue output (DATAQ model DI-40). A needle probe (0.8 mm in diameter; Vasamedic, St Paul, MN, USA) was gently inserted into the central area of the tumour through an 18G catheter (Baxterm Baxter Health Care Corporation, Deerfield, IL, USA) that was preplaced to the desired intratumoral location, using techniques previously described (Acker et al, 1990). A 1.0-mm-diameter probe was placed into a small skin incision over the gastrocnemius muscle proximal to the tumour to measure muscle blood flow. Probe tips were moistened with heparinized saline (20 U $ml^{-1}$) before insertion into tissue. All probes were secured by taping them to a Plexiglas stage, which maintained them in a stable position without pressure on the tissue. During experiments, the probes were sequentially connected to the LaserFlow device for data recording (10–15 s for each probe location). The resultant data are reported as relative changes in flow, as this device is not calibrated for perfusion measurements in tumour tissues (Song et al, 1987).

### Arteriolar diameter measurements

Anaesthetized rats with window chambers were placed in right lateral recumbency on a microscope stage (Zeiss Photomicroscope III, Carl Zeiss, New York, NY, USA). The window preparations were observed by transillumination with a 40-W tungsten light source at × 200. Tumour feeding arterioles were visualized by examining the subcutaneous vascular bed that resides beneath the tumour. We have defined the criteria for determining arterioles in this model system as: (1) visualization of smooth muscle wall; (2) straight path with few branches; (3) direct observation of divergent flow; and (4) for tumour arterioles, divergent flow must traverse into the tumour mass (Dewhirst et al, 1994). Images of focused vessels were captured with a video camera (CCD-72, Dage MTI, Michigan City, IN, USA) and recorded on SVHS videotape (Model BV-1000, Mitsubishi Electronics, Japan). A video-timer signal (ForA., Lid. Model VTG-55, Los Angeles, CA, USA) was superimposed on the images for record keeping. For arteriolar diameter measurements, the videotaped images were analysed with an Image Shearing Monitor (Model 907, Instrumentation for Physiology & Medicine, San Diego, CA, USA) as previously described (Dewhirst et al, 1989).

### Analysis for vasomotion

For the first 10 min after DEA/NO administration, arteriolar diameters were measured every 30 s and for all maxima and minima of the vasomotion cycles. Relative changes in arteriolar diameter were plotted as a function of time of observation. Vasomotion cycles were defined as a diameter change between sequential maxima or minima (peak to peak or valley to valley) that were larger than a 'threshold' value. For arterioles with baseline diameters less than 20 μm, this was defined as > 5% change in diameter. For arterioles with baseline diameters greater than 20 μm, the threshold was set at 2 μm. To quantify the difference in vasomotor frequency between tumour and granulating tissue arterioles, we compared the following parameters: (1) number of cycles during the 10-min period of observation and (2) frequency of vasomotion, defined as the number of cycles divided by the overall time of observation.

### Tumour oxygenation measurements

Recessed-tip oxygen microelectrodes, with 6 to 20-μm-diameter tips were manufactured in our laboratory using the method of Linsenmeier and Yancey (1987). The cathodes were coated with a gas-permeable membrane (Rhoplex; Rohm and Haas, Philadelphia, PA, USA) to prevent possible electrode poisoning. Electrode current was measured with a microsensor (Chemical Microsensor, Model 1201; Diamond General, Ann Arbor, MI, USA) and the output was connected to a PC computer equipped with the CODAS software described above. Each electrode was calibrated against four standard gases two or three times before use in vivo in order to determine linearity and reproducibility of response; calibrations were also performed after experimentation to again confirm linearity. Conversion of current to oxygen partial pressure ($pO_2$) was accomplished using the in vitro calibration line, which also included an in vivo value recorded in the tumour after death (Dewhirst et al, 1992*b*). Intratumoral measurements were made by

 © Cancer Research Campaign 1997

inserting the electrode into the tumour mass with a micromanipulator and leaving the electrode in place for the duration of the experimental protocol.

$pO_2$ measurements were performed in seven leg tumour-bearing rats following a single dose of 1000 nmol $kg^{-1}$. In five rats, two 0.4-mm-diameter needle LDF probes were also inserted into the tumour before the oxygen electrode. The LDF was measured continuously throughout the experiments, using the Oxford Array System (Oxford Optronix, Oxford, UK).

### Experimental protocols

Four groups of rats were used to study: (1) blood flow in leg tumours and adjacent muscle with LDF ($n$ = 13); (2) changes in diameter of tumour-feeding arterioles ($n$ = 12); (3) changes in arterioles of normal granulating tissue in window chambers without tumour ($n$ = 5); and (4) changes in tumour oxygenation/LDF in leg tumours after DEA/NO administration ($n$ = 7).

Animals were anaesthetized with pentobarbital sodium (Abbott Laboratories, North Chicago, IL, USA) intraperitoneally at a dose of 40 mg $kg^{-1}$. Depth of anaesthesia was monitored by evaluation of spontaneous animal movement and by response to stimuli (withdrawal reflex and/or blink reflexes). Redosing of anaesthetic (25% of initial dose) was given 10 min before starting experiments, when required. The right femoral artery was cannulated and connected to the data acquisition system for mean arterial pressure (MAP) and heart rate measurements. The femoral vein was cannulated for i.v. access. The animals were kept warm on a water thermoblanket (American Pharmaseal, Valencia, CA, USA) or a Homeothermic Blanket Control Unit (Harvard Apparatus Limited, Edenbridge, KY, USA) throughout experiments. Baseline parameters were recorded for at least 10 min before injection of test solutions.

A previous report (Diodati et al, 1993) and our pilot experiments demonstrated that the cardiovascular effect of DEA/NO lasted less than 30 min. Therefore, in order to minimize animal use, each animal was treated with different doses (three or four injections) and the order of dosing was variable. At least 30 min was allowed between the end of recording data for one dose and measurement of baseline for the next test dose. Additional doses were only given when MAP returned to within 5% of pretreatment values.

Because of its short half-life at physiological pH and temperature (Diodati et al, 1993), the effect of delayed administration on NO release from DEA/NO was tested. Successive 1000 nmol $kg^{-1}$ doses were injected in one window tumour-bearing rat following 10-, 20- and 30-min delays after dilution with PBS. The diluted DEA/NO solution was kept at 37°C until injection.

For blood flow studies, LDF recording at the tumour centre was continued from the start of injection to 1 min after the end of injection for each test solution. Subsequent LDF measurements were recorded (for 10–15 s) in the tumour and in muscle at 1, 2 and 5 min after injection and then at 5-min intervals up to 35 min after injection.

For the arteriolar diameter study, images for baseline data were recorded for 1 min followed by continuous recording from the beginning of injection to 10 min after the end of DEA/NO injection. After that recordings were made every 5 min up to 35 min after injection.

For the oxygenation/LDF study, the oxygen microelectrode and two needle laser Doppler probes were placed in the tumour and remained at the same sites for the entire experiment. Intratumoral $pO_2$ was recorded several times during the 5–15 min before DEA/NO administration to test electrode stability. The $pO_2$ was continuously recorded from 20 s before DEA/NO infusion until 2 min after the end of the infusion. Then $pO_2$ was measured for 15–20 s every minute until 5 min after infusion. Thereafter $pO_2$ was recorded at 5 min intervals for the next 25 min. LDF in this study was recorded continuously throughout the experiments. These measurements were only carried out at a DEA/NO dose of 1000 nmol $kg^{-1}$.

**Table 1** Baseline means for MAP and heart rate for different doses and groups

| Dose (nmol $kg^{-1}$) | MAP (mmHg) | | Heart rate (beats $min^{-1}$) | |
|---|---|---|---|---|
| | Window chamber with tumour | Window chamber without tumour | Window chamber with tumour | Window chamber without tumour |
| Decomposed DEA/NO | 109<br>(96–129)[a]<br>$n$ = 6 | | 320<br>(113–342)<br>$n$ = 6 | |
| 10 | 110<br>(95–118)<br>$n$ = 6 | | 336<br>(305–394)<br>$n$ = 6 | |
| 50 | 108<br>(103–124)<br>$n$ = 5 | | 363<br>(349–413)<br>$n$ = 5 | |
| 100 | 106<br>(94–120)<br>$n$ = 5 | 114<br>(83–118)<br>$n$ = 4 | 333<br>(295–409)<br>$n$ = 5 | 355<br>(304–429)<br>$n$ = 4 |
| 500 | 109<br>(96–114)<br>$n$ = 6 | 114<br>(103–123)<br>$n$ = 5 | 326<br>(290–362)<br>$n$ = 6 | 401<br>(355–414)<br>$n$ = 5 |
| 1000 | 108<br>(105–118)<br>$n$ = 5 | 113<br>(96–123)<br>$n$ = 5 | 345<br>(308–410)<br>n = 5 | 355<br>(341–428)<br>$n$ = 5 |

[a]The numbers in parentheses indicate 95% confidence limits.

© Cancer Research Campaign 1997

**Figure 1** Effects of DEA/NO on mean arterial pressure (MAP) (**A**) and heart rate (**B**) in rats bearing dorsal skinfold window chambers with R3230Ac mammary carcinoma. There were no changes in MAP or heart rate after administration of decomposed DEA/NO (—○—), 10 or 50 nmol $kg^{-1}$ (not shown). DEA/NO caused a rapid decrease in MAP at doses ≥ 100 nmol $kg^{-1}$. The degree of hypotension increased with dose ($P < 0.001$) (100 nmol $kg^{-1}$, –■–; 500 nmol $kg^{-1}$, –△–; 1000 nmol $kg^{-1}$, –▽–). DEA/NO at doses ≥ 100 nmol $kg^{-1}$ also reduced heart rate. Twenty minutes after DEA/NO injection both MAP and heart rate were close to baseline, so the data after 20 min are not shown for clarity. Each point with error bars represents the mean and 95% confidence limits of five or six rats.

**Figure 2** Relative changes in blood flow in leg muscle near tumour (**A**) and in the subcutaneously implanted tumour (**B**) after administration of DEA/NO as measured by laser Doppler flowmetry (LDF). There were no significant changes in LDF for control solutions of decomposed DEA/NO (—○—) or PBS or low doses of DEA/NO (not shown) in either muscle or tumour. Muscle blood flow decreased after DEA/NO at the two highest doses (**A**; see text for details). DEA/NO at ≥ 100 nmol $kg^{-1}$ (100 nmol $kg^{-1}$, –■–; 500 nmol $kg^{-1}$, –△–; 1000 nmol $kg^{-1}$, –▽–) significantly decreased tumour blood flow (**B**). All changes in LDF were not significant 20 min after DEA/NO injection, so the data after 20 min are not shown. Each point with error bars represents the mean and 95% confidence limits. For tumour blood flow data, $n$ = 6–8 for 100 and 500 nmol $kg^{-1}$ doses; for 1000 nmol $kg^{-1}$ dose, data include rats from $pO_2$/LDF experiments ($n$ = 14).

### STATISTICS

We analysed changes over time seen in MAP, heart rate and blood flow using the logarithm of measurements divided by their corresponding baseline values. In the arteriolar diameter study, several vessels appeared to close completely at some measurements times, leading to relative diameters equal to zero. To avoid problems caused by taking the logarithm of zero values, we analysed relative change in cross-sectional area, defined as the difference between the lumen area at some time points after infusion and the baseline area divided by the baseline area. The cross-sectional area was estimated by multiplying the numerical constant $\pi$ times one-quarter the measured vessel's diameter squared.

The data set contains correlated measurements, as animals contributed multiple data by way of measurements of several vessels, at several DEA/NO doses, and over time. The statistical analyses accounted for the correlation of within-subject data by including a subject random effect in the SAS MIXED procedure (SAS Institute 1992). Because most profiles appeared highly non-linear, we report statistical comparisons carried out at several key times after the infusion (namely 0.5, 1, 2, 5, 10, and 20 min). We used orthogonal contrasts with an approximate $F$-test to examine the statistical significance of linear dose effects, such as whether increasing the dose of DEA/NO produced effects of greater (or lesser) magnitude. We adjusted for differences in baseline area when comparing lumen areas. The significance of changes in $pO_2$ from baseline was based on the Wilcoxon signed-rank test. Results of two-sided statistical significance tests were deemed significant at the 0.05 level.

### RESULTS

### Effect of time between DEA/NO dilution and injection

DEA/NO rapidly releases NO once it is dissolved in a medium of physiological pH and temperature; its half-life is reported to be 2.1 min (Diodati et al, 1993). In one tumour window chamber-bearing rat, we tested the effect of time delay after dilution of stock DEA/NO into PBS (pH 7.4 prewarmed at 37°C) at a dose of 1000 nmol $kg^{-1}$. Infusion after a 10-min delay caused a marked drop in arterial pressure and concomitant changes in arteriolar

 © Cancer Research Campaign 1997

**Figure 3** Relative changes in arteriolar cross-sectional area in tumour and normal granulating tissue in the dorsal skinfold window chambers after DEA/NO administration. In normal granulating tissue (**A**), arterioles dilated immediately after DEA/NO administration at doses ≥ 100 nmol kg⁻¹ (100 nmol kg⁻¹, -■-; 500 nmol kg⁻¹, -△-; 1000 nmol kg⁻¹, -▽-). Vasodilation lasted for 2–3 min. After that there was a trend towards vasoconstriction but the changes were not significant compared with baseline. There were no significant changes in tumour-feeding arterioles (**B**) after injection of decomposed DEA/NO (—○—) or at 10 or 50 nmol kg⁻¹ (not shown). Significant vasoconstriction occurred immediately after DEA/NO injection at doses ≥ 100 nmol kg⁻¹. Arteriolar diameter gradually returned to baseline as blood pressure recovered. No significant differences were observed after 20 min. Each point with bars represents the mean and 95% confidence limits for four to six rats diameter, but 20- and 30-min delays diminished the hypotensive and microvascular vasoactive effects of DEA/NO in a time-dependent pattern (data not shown). An incubation of PBS-diluted solution at 37°C for 1.5 h completely abolished the cardiovascular effects of DEA/NO. For this reason, the stock drug of DEA/NO was diluted to PBS quickly using a one-step method, and the time interval between dilution and completion of the i.v. injection was kept to no more than 1 min for all experiments.

### Arterial pressure and heart rate

Baseline MAP and heart rates for all test DEA/NO doses in the multidose-treated rats with leg tumours and window chambers (with or without tumour) as well as for single dose-treated rats in the $pO_2$ study were similar, averaging around 100 mmHg and 350 beats min⁻¹ respectively. Table 1 summarizes the MAP and heart rate baseline values in the groups of rats bearing window chambers with or without tumour.

Figure 1 shows the effects of DEA/NO infusion on MAP and heart rate in the rats bearing window chambers with tumour. MAP dropped quickly in the animals receiving at least 100 nmol kg⁻¹

**Figure 4** Examples of arteriolar vasomotion in tumour window chambers in two experiments during hypotension after DEA/NO at the dose of 500 nmol kg⁻¹ (top) and 1000 nmol kg⁻¹ (bottom). The thick line with symbols in each panel represents MAP changes and lines without symbols indicate diameter changes of individual arterioles. Vasomotor activity seemed to be synchronic for parent and daughter arterioles

DEA/NO (Figure 1A), falling to 35–40% of baseline by 30 s after infusion among animals receiving 500 and 1000 nmol kg⁻¹ DEA/NO. Two minutes after infusion, MAP was around 60% of baseline among animals receiving these two highest doses. MAP continued returning to baseline in these animals, reaching around 82% of baseline 10 min after drug infusion. Twenty minutes after infusion, MAP was around 94% of baseline at these two highest doses of DEA/NO. Control solution and low doses of DEA/NO (10, 50 nmol kg⁻¹, data not shown) did not significantly change MAP. The degree of hypotension increased with dose ($P < 0.001$) at each of the first five time-points examined (0.5, 1, 2, 5, and 10 min after DEA/NO infusion) but not at 20 min ($P = 0.174$). All other experimental groups showed similar changes.

There were no significant changes in heart rate in controls or at low doses of DEA/NO (≤50 nmol kg⁻¹, data not shown). Higher doses of DEA/NO produced greater reduction in heart rate among the tumour window experiments ($P < 0.01$) (Figure 1B) at 0.5, 1, 2 and 5 min, but not 10 or 20 min after injection. In the tumour window chamber experiments, 1000 nmol kg⁻¹ DEA/NO produced significant heart rate reduction to around 85% of baseline at 0.5, 1, 2, 5 and 10 min after injection ($P < 0.01$ at each time examined). Similar changes in heart rate were observed in all other groups.

### Tumour blood flow

No change in blood flow was observed after administration of PBS or decomposed DEA/NO in either muscle or tumour (Figure 2).

Blood flow in normal muscle decreased among animals receiving 500 and 1000 nmol kg⁻¹ DEA/NO, although not immediately (Figure 2A). In fact, blood flow increased initially among some animals receiving the higher doses of DEA/NO, leading to quite heterogeneous relative-flow measurements for about the first 5 min.

© Cancer Research Campaign 1997

**Figure 5** Comparison of vasomotor activity between tumour (▤) and normal (□) arterioles after administration of 500 nmol kg$^{-1}$ DEA/NO. The vasomotor frequency and the total number of cycles in the first 10 min after DEA/NO administration were significantly greater in tumour (17 arterioles in five window chambers) than in normal tissues (eight arterioles in five window chambers) (*$P < 0.01$)

The blood flow was 79% ($P = 0.012$) and 70% ($P < 0.001$) of baseline 10 min after injection in the 500 and 1000 nmol kg$^{-1}$ groups respectively. Even at 20 min, blood flow was still only 74% ($P = 0.038$) of baseline among animals receiving 1000 nmol kg$^{-1}$ DEA/NO. DEA/NO doses of 100 nmol kg$^{-1}$ and less did not produce significant blood flow changes in the muscle at any time examined.

In tumours, the duration and degree of blood flow reduction increased with DEA/NO dose (Figure 2B). Tumour blood flow was 75% ($P = 0.023$) and 55% ($P < 0.001$) of baseline 30 s after injection of 500 and 1000 nmol kg$^{-1}$ DEA/NO respectively. Tumour blood flow was between 57% and 75% of baseline at 1, 2 and 5 min after injection of these two highest DEA/NO doses ($P < 0.001$). Tumour blood flow was still significantly reduced after DEA/NO at these doses 10 min after the injection (75% and 83%, $P = 0.003$ and $P = 0.013$ respectively). The animals receiving 100 nmol kg$^{-1}$, on the other hand, experienced a significant flow reduction to 84% ($P = 0.039$) of baseline 2 min after injection; the changes were not significantly different from zero at the other times checked. Blood flow in tumours essentially returned to baseline at all doses examined 20 min after injection of the drug, except for animals that received 500 nmol kg$^{-1}$ (relative flow 84%; $P = 0.032$).

### Arteriolar cross-sectional area

The vasoactive response of arterioles to DEA/NO infusion in normal granulating tissue was short-lived (Figure 3A). An increase in diameter was observed immediately after injection of DEA/NO at doses ≥ 100 nmol kg$^{-1}$ ($P \leq 0.01$ at 30 s and $P \leq 0.03$ at 1 min). Significant vasodilation was still evident at 2 min only in animals treated at 1000 nmol kg$^{-1}$ ($P = 0.01$). Maximal vasodilation ranged between 30% and 66% of baseline cross-sectional area and lasted 2–3 min. At the dose of 1000 nmol kg$^{-1}$, DEA/NO caused 66% vasodilation 30 s after injection ($P = 0.002$) and the lumen area was still 64% larger than baseline at 1 min ($P = 0.009$). Although there was a trend towards vasconstriction during recovery of MAP, the lumen areas were not statistically different from their baseline values. The cross-sectional areas of arterioles were not significantly different from baseline at 10 and 20 min and thereafter.

There were no significant changes in tumour arteriolar cross-sectional area after injection of decomposed DEA/NO (Figure 3B). Low doses (10 and 50 nmol kg$^{-1}$) of DEA/NO had no significant effect on tumour arteriolar diameter (data not shown). Arteriolar vessels constricted significantly in animals treated at doses ≥ 100 nmol kg$^{-1}$ at 2 and 5 min after the infusion ($P < 0.023$). Tumour arterioles in animals treated at 500 and 1000 nmol kg$^{-1}$ DEA/NO remained constricted at 10 min at these two highest doses ($P \leq 0.023$). By 20 min after injection, no significant changes from baseline were observed. The duration of vasoconstriction in tumours seemed somewhat longer at the two highest doses (500 and 1000 nmol kg$^{-1}$) compared with 100 nmol kg$^{-1}$. Whereas lumen area of tumour arterioles in animals treated with 100 nmol kg$^{-1}$ DEA/NO was around 74% of baseline at 1 and 2 min after injection ($P = 0.002$ and $P = 0.004$ respectively) and 93% of baseline at 10 min ($P = 0.204$), in animals treated at the dose of 1000 nmol kg$^{-1}$ DEA/NO arterioles had narrowed significantly 2 min after DEA/NO injection (about 80% of baseline; $P = 0.023$) and were still only 87% of baseline at 10 min ($P = 0.023$). The difference in relative cross-sectional area between the 100 nmol kg$^{-1}$ group and the animals treated at the two highest doses, however, was not significant at 10 min ($P = 0.080$). However, the animals treated with doses of at least 100 nmol kg$^{-1}$ did experience significantly greater tumour arteriole vasoconstriction than the animals treated at the control (Figure 3B) or lower doses (10 and 50 nmol kg$^{-1}$, data not shown) at 2, 5 and 10 min after injection ($P \leq 0.012$).

As can be seen by comparing the two graphs in Figure 3, there were substantial differences between tumour and normal tissue arterioles in response to DEA/NO at doses ≥ 100 nmol kg$^{-1}$. Whereas normal arterioles dilated just after the infusion of DEA/NO, tumour-feeding arterioles constricted by up to 40%. The relative changes in lumen area were significantly different in tumour-feeding arterioles compared with normal arterioles at 0.5, 1, 2 and 5 min after infusion ($P < 0.05$).

### Arteriolar vasomotion

In more than half the animals treated with higher doses of DEA/NO, there were rhythmic oscillations in tumour arteriolar diameter that were most pronounced during the period of hypotension and gradually recovered as MAP returned towards baseline. Figure 4 demonstrates examples of such arteriolar vasomotion in two experiments at doses of 500 and 1000 nmol kg$^{-1}$. In a few cases arterioles completely constricted for intervals of a few seconds at a time. The vasomotion for parent and daughter arterioles was synchronous. A reduction in arteriolar blood flow rate was also observed during hypotension. Intermittent reversed flow and temporary stasis were occasionally observed, even while arterioles were dilated.

At a dose of 500 nmol kg$^{-1}$, arteriolar vasomotion in normal granulating tissue was less commonly observed than in tumour-feeding arterioles. Vasomotor frequency averaged 2–3 times higher in tumour arterioles than in normal arterioles at this dose

© Cancer Research Campaign 1997

**Figure 6** Effect of DEA/NO (1000 nmol $kg^{-1}$) administration on intratumoral $pO_2$ in seven leg tumour-bearing rats ($n$ = 4 at 3 and 4 min and $n$ = 7 at all other time points) (**A**). In a subgroup of five rats, tumour blood flow was measured simultaneously with $pO_2$ using laser Doppler flowmetry throughout the experiments (**B**). Immediate drops in tumour $pO_2$ and tumour blood flow after DEA/NO were almost synchronous with the change in mean arterial pressure (**C**). There were no significant differences in MAP, tumour LDF or $pO_2$ compared with baseline 20 min after DEA/NO infusion (not shown). Each point represents the mean ± 1 s.e.m.

($P$ < 0.01; Figure 5). The total number of cycles over the first 10 min was also higher in tumour than in granulating tissue ($P$ < 0.01; Figure 5). Interestingly, there was no significant difference in these parameters of vasomotor activity at a dose of 1000 nmol $kg^{-1}$, as the vasoactivity of normal arterioles increased to the level shown by the tumour arterioles. The number of cycles for the first 10 min was 4.2 ± 0.8 for the tumour arterioles and 4.3 ± 0.6 for the normal arterioles. Similarly, the frequency was 0.58 ± 0.06 c.p.m. and 0.75 ± 0.12 c.p.m. for the tumour and normal arterioles respectively (mean ± s.e.m.; $n$ = 5 rats in each group).

### Intratumoral $pO_2$

The effects of 1000 nmol $kg^{-1}$ of DEA/NO on tumour oxygenation were determined in seven experiments. $pO_2$ averaged 10 mmHg before drug administration (Figure 6A). Immediately after DEA/NO administration, intratumoral $pO_2$ dropped by an average of 8 mmHg and was significantly lower than baseline for 10 min ($P$ < 0.05). Tumour $pO_2$ was not significantly different from baseline 15 or 20 min after DEA/NO administration. The decrease in $pO_2$ occurred simultaneously with decreases in tumour blood flow (Figure 6B) and MAP (Figure 6C).

### DISCUSSION

The results of this study demonstrate that DEA/NO decreases MAP, heart rate and tumour blood flow as well as tumour arteriolar diameter. In general, effects were seen at doses of at least 100 nmol $kg^{-1}$, and higher doses were associated with larger effects. At the dose of 1000 nmol $kg^{-1}$ DEA/NO, the drop in blood flow was accompanied by a substantial reduction in tumour $pO_2$. The decreases in MAP, tumour blood flow and $pO_2$ occurred synchronously. These results suggest that administration of this type of vasodilator will result in decreased peripheral perfusion pressure, which leads to reduction in blood flow and oxygenation in tumours. Thus, the primary hypothesis of the study was proven.

The effect of DEA/NO on systemic arterial pressure observed in this study was consistent with previous reports (Diodati et al, 1993). As predicted from its rate of spontaneous NO release, DEA/NO is a short-acting vasodilator with a duration of action similar to that of nitroprusside (Prescott et al, 1992). At doses ≥ 100 nmol $kg^{-1}$, it caused significant reductions in MAP and heart rate that were dose dependent. Most other hypotensive agents cause compensatory tachycardia as a result of baroreceptor reflexes; thus, the negative chronotropic effect of this agent is somewhat unique and is probably related to direct effects of NO on cardiac muscle contractility (Roberts et al, 1992).

The reduction in tumour blood flow after DEA/NO administration is probably due to two factors. First, vasodilatory effects in normal arterioles in window chambers, combined with lack of vasodilation in tumour arterioles at the time of hypotension, are direct evidence for a vascular steal phenomenon, at least in the window chamber preparation. In fact, the classic definition of this phenomenon includes these features (Dewhirst et al, 1992*a*). However, a vascular steal phenomenon is not a prerequisite for preferential reduction in tumour blood flow. We have previously reported that the peripheral vasodilator hydralazine induces preferential reduction in tumour blood flow in the window chamber tumour without concomitant normal tissue vasodilation in the tumour bed. In this case, the predominant mechanism involved is probably relatively higher flow resistance in tumours, which is exacerbated when the driving pressure is reduced (Dewhirst et al, 1994; Sevick and Jain, 1989). We suspect that the sustained reduction in tumour blood flow that persists beyond the normal tissue vasodilatory stage with DEA/NO is probably due to this second mechanism, as the hypotensive effects lasted longer than the vasodilatory effects in normal granulating subcutaneous tissue that constituted the tumour bed. The second mechanism is probably predominant in the leg tumours as well, because we did not see any evidence for improvement in normal muscle blood flow that was near tumour during the period when tumour blood flow was reduced. If a vascular steal phenomenon had occurred in that site, one would have expected that muscle blood flow surrounding the tumour would have been increased as blood would have been shunted away from the tumour and into the surrounding normal muscle.

© Cancer Research Campaign 1997

Administration of DEA/NO at higher doses led to reduction in normal muscle blood flow, although not to the same extent as in tumour tissue. There is precedence in the literature to suggest that similar effects are seen in bone marrow. Liebmann et al (1994), found that DEA/NO and NOS inhibition with $N^G$-nitro-L-arginine exerted radioprotection against whole-body irradiation in C3H mice. They speculated that alterations in regional blood flow and oxygenation may underlie the changes in radiation tolerance.

It is interesting to compare the effects of DEA/NO, an NO donor, with inhibitors of NO synthase, because the net effect of both manipulations is a reduction in tumour blood flow, even though they produce opposite effects in terms of affecting NO levels. Several previous reports have shown that inhibition of NOS by analogues of L-arginine leads to tumour blood flow reduction (Andrade et al, 1992; Wood et al, 1994 *a,b*; Meyer et al, 1995). In this case, the vasoconstrictive effects of NO synthase inhibition are probably responsible for the effects, as such treatment actually induces mild hypertension. Again, the drop in tumour blood flow is probably exacerbated in tumours because of relatively high flow resistance (Sevick and Jain, 1989). Although rapid release of NO from DEA/NO causes substantial vasodilation in normal tissue and reduction in systemic resistance, tumour blood perfusion decreases because of the significant hypotensive effect. Systemic administration of such a potent and short-acting NO donor did not dilate tumour-feeding arterioles, instead it caused vasoconstriction, which probably reflects differences in NO regulation mechanisms between tumour and normal microvasculature. It may be that tumour arterioles, unlike normal arterioles, are already maximally dilated because of excessive production of NO in solid tumour (Doi et al, 1996) and thus lack a vasodilatory response to exogenous NO. In addition, increased interstitial pressure in tumour mass might result in a 'passive collapse' of tumour vessels when intravascular pressure decreases during DEA/NO-induced hypotension.

The induction of vasomotor responses in tumour arterioles following DEA/NO infusion is intriguing. It is possible that the vasomotion may reflect local autoregulatory responses to DEA/NO-induced hypotension, lowered TBF and hypoxia. Induction of vasomotor activity in normal arterioles has previously been reported in response to reduction in systemic pressure as well as reduction in oxygenation (Bertuglia et al, 1991; Vollmar et al, 1994). However, the effect seems to be controlled at the tissue level, rather than by systemic factors. Evidence for local control comes from experiments in which local compression of the femoral artery led to increased arteriolar vasomotion in dependent muscle tissue (Schmidt et al, 1992). The effect induced by hypoxia has been shown to be caused by enhanced endothelial cell production of endothelin, which is also consistent with a local tissue effect (Kourembanas et al, 1991). Our current results are relevant to the notion that the vasomotor activity is locally mediated in tumours as well. The finding that the vasoactive response in tumour is exaggerated compared with that in normal arterioles at a dose of 500 nmol $kg^{-1}$ suggests that the vasoactivity threshold for tumour arterioles may be lower than that for normal arterioles. This is consistent with the findings of Kennovin et al (1994), who demonstrated that vasomotor activity in excised arteries that had been feeding implanted tumours in the epigastric pedicle had increased vasomotor activity, compared with normal control arterioles.

It has been reported previously that administration of another NO donor drug, SIN-1, leads to improved energy status and a threefold improvement in radiation response of the transplanted SCCVII/Ha tumour of C3H mice (Wood et al, 1993). This combination of effects would lead one to believe that SIN-1 improves tumour blood flow and hence oxygenation. However, if the physiological effects of SIN-1 are similar to those seen with DEA/NO, the net result would be to reduce tumour blood flow and oxygenation rather than increase it. However, SIN-1 also releases peroxynitrite and hydroxyl radicals upon degradation, which may have other, as yet to be defined, physiological effects (Feelisch and Stamler, 1996). Another mechanism of enhanced radiation response with NO is the direct radiosensitizing effect. Like oxygen, NO can bind to carbon-centred radicals and prevent repair of radiation-induced damage and increase the cytotoxicity of radiation (Howard-Flanders, 1957). Mitchell et al (1993) found that DEA/NO is capable of sensitizing hypoxic, not aerobic, V79 cells with enhancement ratios ranging from 1.1 to 2.4 at 0.1–1 mM concentration. Thus, the radiosensitization effect of NO might be independent of changes in tumour blood flow or oxygenation.

A brief justification needs to be made for the use of the stationary recessed-tip microelectrodes that were used for the oxygen measurements. The reason that we did not use the Eppendorf system, which is available to us, is twofold: (1) we have found that performance of repeated measurements of $pO_2$ in the same tumour with the Eppendorf system leads to hypoxia from tissue damage and (2) the time course over which the hypoxia was expected to occur would preclude measurements with a moving electrode device. There is potential for stationary electrodes to consume oxygen, thereby leading to artifactually low $pO_2$ readings. We do not believe that this happened in this case, because the $pO_2$ readings in both cases were quite stable for the 5–15-min period before DEA/NO administration. Secondly, the recessed-tip design minimizes consumption from this type of electrode (Schneiderman and Goldstick, 1978).

### ACKNOWLEDGEMENTS

The authors thank Dr LK Keefer for his gift of DEA/NO. This work is supported by grants from Apex Bioscience, and NIH/NCI CA 40355 and NIH/NEI EY06516 (RDB).

### REFERENCES

Acker JC, Dewhirst MW, Honore GM, Samulski TV, Tucker JA and Oleson JR (1990) Blood perfusion measurements in human tumours; evaluation of laser Doppler methods. *Int J Hyperthermia* **6**: 287–304

Andrade SP, Hart IR and Piper PJ (1992) Inhibitors of nitric oxide synthase selectively reduce flow in tumour-associated neovasculature. *Br J Pharmacol* **107**: 1092–1095

Bertuglia S, Colantuoni A, Coppini G and Intaglietta M (1991) Hypoxia or hyperoxia-induced changes in arteriolar vasomotion in skeletal muscle microcirculation. *Am J Physiol* **260**: H362–372

Dewhirst MW, Tso CY, Oliver R, Gustafson CS, Secomb TW and Gross JF (1989) Morphologic and hemodynamic comparison of tumour and healing normal tissue microvasculature. *Int J Radiat Oncol Biol Phys* **17**: 91–99

Dewhirst MW, Vinuya RZ, Ong ET, Klitzman B, Rosner G, Secomb TW and Gross JF (1992*a*) Effects of bradykinin on the hemodynamics of tumour and granulating normal tissue microvasculature. *Radiat Res* **130**: 345–354

Dewhirst MW, Ong ET, Klitzman B, Secomb TW, Vinuya RZ, Dodge R, Brizel D and Gross JF (1992*b*) Perivascular oxygen tensions in a transplantable mammary tumour growing in a dorsal flap window chamber. *Radiat Res* **130**: 171–182

Dewhirst MW, Madwed D, Meyer RE, Ong ET, Klitzman B, Rosner GL, Dodge R (1994) Reduction in tumour blood flow in skin flap tumour after hydralazine is not due to a vascular steal phenomenon. *Radiat Oncol Invest* **1**: 270–278

Diodati JG, Quyyumi AA and Keefer LK (1993) Complexes of nitric oxide with nucleophiles as agents for the controlled biological release of nitric oxide: hemodynamic effect in the rabbit. *J Cardiovasc Pharmacol* **22**: 287–292

© Cancer Research Campaign 1997

Doi K, Akaike T, Horie H, Naguchi Y, Fujii S, Beppu T, Ogawa M and Maeda H (1996) Excessive production of nitric oxide in rat solid tumour and its implication in rapid tumour growth. *Cancer* **77** (suppl 8): 1598–1604

Feelisch M and Stamler JS (1996) Donors of nitrogen oxides. In *Methods in Nitric Oxide Res*. Feelisch M and Stamler JS (eds), pp. 71–118. John Wiley: West Sussex, UK

Howard-Flanders P (1957) Effect of nitric oxide on the radiosensitivity of bacteria. *Nature* **180**: 1191–1192

Kennovin GD, Flitney FW and Hirst DG (1994) 'Upstream' modification of vasoconstrictor response in rat epigastric artery supplying an implanted tumour. In *Oxygen Transport to Tissue* XV, Vaupel P (ed), pp. 411–416. Plenum Press: New York

Kourembanas S, Marsden PA, Mcquillan LP and Faller DV (1991) Hypoxia induces endothelin gene expression and secretion in cultured human endothelium. *J Clin Invest* **88**: 1054–1057

Liebmann J, Deluca AM, Coffin D, Keefer LK, Venzon D, Wink DA and Mitchell JB (1994) In vivo radiation protection by nitric oxide modulation. *Cancer Res* **54**: 3365–3368

Linsenmeier RA and Yancey CM (1987) Improved fabrication of double-barreled recessed cathode $O_2$ microelectrodes. *J Appl Physiol* **63**: 2554–2557

Meyer RE, Shan S, De Angelo J, Dodge RK, Bonaventura J, Ong ET, Dewhirst MW (1995) Nitric oxide synthase inhibition irreversibly decreases perfusion in the R3230AC rat mammary carcinoma. *B J Cancer* **71**: 1169–1174

Mitchell JB, Wink DA, Degraff W, Gamson J, Keefer LK and Krishna MC (1993) Hypoxic mammalian cell radiosensitization by nitric oxide. *Cancer Res* **53**: 5845–5848

Papenfuss D, Gross JF, Intaglietta M and Treese FA (1979) A transparent access chamber for the rat dorsal skin fold. *Microvasc Res* **18**: 311–318

Prescott DM, Samulski TV, Dewhirst MW, Page RL, Thrall DE, Dodge RK and Oleson JR (1992) Use of nitroprusside to increase tissue temperature during local hyperthermia in normal and tumour-bearing dogs. *Int J Radiat Oncol Biol Phys* **23**: 377–385

Roberts AB, Vodovotz Y, Roche NS, Sporn MB and Nathan CF (1992) Role of nitric oxide in antagonistic effects of transforming growth factor-beta and interleukin-1 beta on the beating rate of cultured cardiac myocytes. *Mol Endocrinol* **6**: 1921–1930

SAS Institute (1992) SAS Technical Report P-229, *SAS/STAT Software: Changes and Enhancements*, Release 6.07, Chapter 16. SAS Institute: Cary, NC

Schmidt JA, Intaglietta M and Borgstrom P (1992) Periodic hemodynamics in skeletal muscle during local arterial pressure reduction. *J Appl Physiol* **73**: 1077–1083

Schneiderman G and Goldstick TK (1978) Oxygen electrode design criteria and performance characteristics: recessed cathode. *J Appl Physiol: Respirat Environ Exercise Physiol* **45**: 145–154

Sevick EM and Jain RK (1989) Geometric resistance to blood flow in solid tumours perfused ex vivo: Effects of tumour size and perfusion pressure. *Cancer Res* **49**: 3506–3512

Song CW, Makepeace CM, Griffin RJ and Keefer LK (1995) Modification of tumor oxygenation by nitric oxide and inhibitors of nitric oxide synthase. In Tumor Oxygenation, Vaupel PW, Kelleher DK and Gunderoth M (eds), pp.119–124. Gustav Fischer: Stuttgart

Song CW, Rhee JG and Haumschild DJ (1987) Continuous and non-invasive quantification of heat-induced changes in blood flow in the skin and RIF-1 tumour of mice by laser Doppler flowmetry. *Int J Hyperthermia* **3**: 71–77

Vollmar B, Preissler G and Menger MD (1994) Hemorrhagic hypotension induces arteriolar vasomotion and intermittent capillary perfusion in rat pancreas. *Am J Physiol* **267**: H1936–H1940

Wood PJ, Stratford IJ, Adams GE, Szabo C, Thiemermann C and Vane JR (1993) Modification of energy metabolism and radiation response of a murine tumour by changes in nitric oxide availability. *Biochem Biophys Res Comm* **192**: 505–510

Wood PJ, Sansom JM, Butler SA, Stratford IJ, Cole SM, Szabo C, Thiemermann C and Adams GE (1994*a*) Induction of hypoxia in experimental murine tumours by the nitric oxide synthase inhibitor, $N^G$-nitro-L-arginine. *Cancer Res* **54**: 6458–6463

Wood PJ, Sansom JM, Stratford IJ, Adams GE, Szabo C, Thiemermann C, Vane JR (1994*b*) Modification of metabolism of transplantable and spontaneous murine tumours by the nitric oxide synthase inhibitor, nitro-L-arginine. *Int J Radiation Oncol Biol Phys* **29**: 443–447

© Cancer Research Campaign 1997